# Construction and application of a novel respiratory syncytial virus vaccine based on *Bacteroides thetaiotaomicron* outer membrane vesicles

**DOI:** 10.3389/fmicb.2026.1956909

**Published:** 2026-09-03

**Authors:** Xuejiao Li, Xiaomeng Li, Mingyao Jiang, Zhenhua Hao, Yonghe You, Yongsheng Xie, Jinlong Zhang, Leqi Du, Yiding Xu, Fukang Li, Xiaohui Zhao

**Affiliations:** Department of Basic Medicine Science, North Henan Medical University, Xinxiang, Henan, China

**Keywords:** *Bacteroides thetaiotaomicron* outer membrane vesicles, mucosal immunity, OMV-based vaccines, respiratory syncytial virus, vaccine development

## Abstract

Respiratory syncytial virus (RSV) is a leading cause of severe lower respiratory tract infections in infants, young children, immunocompromised adults, and elderly individuals with comorbidities. Its high pathogenicity and associated mortality impose a substantial disease burden on public health. Vaccination represents a key strategy for preventing RSV infection. However, currently approved RSV vaccines exhibit limitations. For instance, Abrysvo and Arexvy pose potential safety risks in clinical use, and the long-term protective efficacy of mRESVIA may wane over time. Therefore, this article proposes and explores a novel RSV vaccine strategy utilizing *Bacteroides thetaiotaomicron* outer membrane vesicles (Bt OMVs) as a delivery platform. This strategy capitalizes on the unique advantages of Bt OMVs, including efficient mucosal delivery, low inherent toxicity, and targeted modulation of T cell homeostasis—specifically enhancing Treg and Th1 responses while suppressing excessive Th2 and Th17 activation, which serves as the core mechanism for preventing vaccine-enhanced disease (VED)—to effectively present the RSV F protein antigen. It offers a novel approach to develop next-generation RSV vaccines that combine durable protection with enhanced clinical safety.

## 1 Introduction

RSV is primarily transmitted via respiratory droplets but can also spread through direct contact with infected secretions or indirect contact via contaminated surfaces and then touching mucosal surfaces (e.g., mouth, nose, eyes) (Kaler et al., 2023). Following host entry, RSV initially replicates within nasopharyngeal epithelial cells, presenting as an upper respiratory tract infection. The virus may subsequently disseminate distally to the lower respiratory tract, potentially triggering bronchiolitis or pneumonia, and in severe cases, respiratory failure (Brasier, 2020). Given its high transmissibility and broad host susceptibility, developing safe and effective RSV vaccines is an urgent global public health priority to alleviate disease burden. However, significant challenges in RSV vaccine development arise from the conformational instability of the RSV F protein and its role in immune evasion (Graham, 2017). In this context, novel vaccine platforms based on OMVs technology are emerging as a promising approach. These leverage the inherent adjuvant properties of OMVs to synergistically stimulate both humoral and cellular immunity (Prior et al., 2021). Nevertheless, classical OMVs derived from pathogenic bacteria face limitations in clinical translation, including inherent pro-inflammatory activity, heterogeneity, and low production yields (Micoli et al., 2024). In contrast, OMVs derived from the human commensal bacterium Bt exhibit a low inflammatory profile and possess immunomodulatory characteristics (Pardue et al., 2024; Durant et al., 2020). These properties position Bt OMVs as a highly promising platform for novel RSV vaccine development. This strategy holds substantial potential for eliciting immunogenicity while circumventing the limitations associated with classical pathogen-derived OMVs, offering a novel pathway towards achieving safe and highly efficacious protection against RSV.

## 2 Current status and challenges in RSV vaccine research

RSV is a leading cause of lower respiratory tract infections, posing a particularly severe threat to the health of infants, young children, and the elderly (Coultas et al., 2019; Li et al., 2022). In infants under 1 year of age, RSV infection characteristically manifests as bronchiolitis, with symptoms including transient fever, cough, rhinorrhea, dyspnea, and decreased feeding (Barr et al., 2019). Clinical presentations in adults are more diverse, ranging from mild cold-like symptoms to severe pneumonia, exacerbation of underlying chronic conditions, and potentially fatal outcomes (Bont et al., 2024). RSV infection also poses a significant risk to immunocompromised populations, such as solid organ transplant recipients (Villanueva et al., 2022). Although infection often requires treatment, current anti-RSV therapeutics exhibit limitations. Palivizumab is restricted to high-risk pediatric populations, with its high cost hindering widespread accessibility (Garegnani et al., 2021). While ribavirin may be effective in select adult cases, it carries risks such as hemolytic reactions and bronchospasm (Mir et al., 2021). Consequently, developing safe and highly effective RSV vaccines represents the fundamental approach to reducing the disease burden. Despite over six decades of research, significant challenges persist in RSV vaccine development. The first candidate, the 1960s formalin-inactivated RSV vaccine (FI-RSV), proved problematic: clinical trials revealed that vaccinated infants experienced enhanced pulmonary immunopathology upon subsequent natural RSV exposure compared to unvaccinated controls—a phenomenon termed vaccine-enhanced disease (VED) that critically impeded subsequent efforts (Li et al., 2016). VED pathogenesis may be associated with vaccine-induced Th2 (T helper 2) and Th17 (T helper 17) cell responses in the lungs, as well as suppression of regulatory T cell (Treg) function (Li et al., 2016; Wang Y. et al., 2023). Furthermore, RSV possesses the ability to evade host innate immunity, and the adaptive immune response it elicits fails to provide sterilizing immunity against reinfection, further complicating vaccine development (Van Royen et al., 2022). Concurrently, the lack of fully permissive animal models that accurately recapitulate human RSV infection limits the evaluation of vaccine efficacy (Xiong et al., 2022). Advances in RSV research have identified the RSV F protein as a promising target due to its highly conserved sequence and the ability of F-induced antibodies to neutralize both RSV-A and RSV-B subtypes (Rezende et al., 2023). Notably, its prefusion conformation (pre-F) exhibits potent immunogenicity (Cox et al., 2023). Based on these characteristics, the vast majority of RSV vaccines currently in preclinical or clinical development are designed using the F protein as the antigen. Although three RSV vaccines targeting RSV pre-F have been approved for marketing, they still exhibit limitations in clinical application (Table 1).

**Table 1:** Comparative analysis of approved RSV vaccines.

RSV vaccines	Type	Target antigen	Target population	Adverse events	Ref.
Abrysvo	Recombinant protein vaccine	Pre-F	Pregnant individuals (32–36 weeks gestation); Adults ≥60 years	A higher incidence of adverse events (including preeclampsia, fetal distress syndrome, preterm birth, and fetal death) was observed compared to placebo.	Patel et al. (2024)
Arexvy	Recombinant protein vaccine	Pre-F	Adults ≥60 years	Common short-term local reactions (e.g., injection site pain); cases of potential cardiovascular risks and serious neurological adverse events have been reported.	Abbasi and Oduoye (2023)
mRESVIA	mRNA vaccine	Pre-F	Adults ≥60 years	Short-term protective efficacy is established, but long-term efficacy data are limited; preliminary data suggest efficacy may decline over time. Durability and long-term safety require ongoing monitoring.	Anastassopoulou et al. (2025)

In summary, RSV vaccine development is progressing rapidly, with multiple candidates showing promise in clinical trials. However, the field continues to face three major challenges: inducing robust and durable protective immune responses, preventing VED following vaccination, and meeting the diverse safety and efficacy needs of different target populations.

## 3 Current status of OMVs transformed into vaccine formulations

Outer membrane vesicles (OMVs) are nanoscale vesicles derived from the outer membrane of Gram-negative bacteria, typically ranging in size from 20 to 300 nm (Avila-Calderón et al., 2021). OMVs carry multiple pathogen-associated molecular patterns (PAMPs), including lipopolysaccharide (LPS), lipoproteins, and peptidoglycan. Specifically, LPS is recognized by Toll-like receptor 4 (TLR4), bacterial lipoproteins engage TLR2 (often forming heterodimers with TLR1 or TLR6), and peptidoglycan derivatives activate nucleotide-binding oligomerization domain (NOD)-like receptors such as NOD1 and NOD2. These receptor-ligand interactions trigger downstream activation of the NF-κB and MAPK signaling pathways, inducing the secretion of pro-inflammatory cytokines and type I interferons, which in turn bridge innate immune activation to the initiation of robust humoral and cellular adaptive immune responses (Cecil et al., 2019; Tong et al., 2024). The lipid bilayer structure of OMVs protects cargo from degradation and facilitates targeted delivery through interactions with bacterial or host cell surface ligands, promoting inter-bacterial and host-bacterial communication (Velimirov and Ranftler, 2018; Bitto et al., 2017). Furthermore, their nanoscale size and vesicular morphology promote widespread *in vivo* distribution, enabling effective vaccine delivery (Tan et al., 2018). The rapid development of high-throughput proteomics and intelligent bioinformatic analysis has greatly improved the ability to comprehensively characterize the protein cargo of OMVs, accurately identify key protective antigenic epitopes, and standardize vaccine quality control, which provides a solid technical foundation for the clinical translation of OMV-based vaccines (Zhang et al., 2025). These properties establish OMVs as natural delivery vehicles with significant potential in vaccine development.

*Helicobacter pylori*, a Gram-negative bacterium primarily colonizing the stomach and duodenum, is associated with gastritis, gastric ulcers, and even gastric cancer (Vale et al., 2024). Evaluation of an OMV-based vaccine in a surgical gastric infection model of *H. pylori* in C57BL/6 mice demonstrated that OMVs induced high levels of antibody responses and activated T cell responses, exhibiting strong immunogenicity (Das et al., 2025). The LPS structure of recombinant *H. pylori* OMVs was engineered, and the carrier advantages of OMVs were leveraged to deliver key *H. pylori* antigenic proteins (UreB, VacA, and CagA), aiming to elicit synergistic vaccine effects (Liu et al., 2025). OMVs derived from *Porphyromonas gingivalis* showed significant immunogenicity and protective efficacy in mouse models, effectively reducing inflammation and destruction of periodontal tissues and preventing the development of periodontitis. This positions them as potential candidate components for periodontal disease vaccine development (Zhang et al., 2020). Irene et al. constructed an OMV-based vaccine containing five lipidated antigens by fusing five *Staphylococcus aureus* (*S. aureus*) protective antigens to lipoprotein sequences and integrating them into *Escherichia coli* OMVs. This vaccine provided nearly 100% protection in a mouse model of *S. aureus* infection (Irene et al., 2019). An OMV-based vaccine incorporating pneumococcal surface protein A (PspA) induced potent humoral and cellular immune responses, providing significant protection against secondary pneumococcal infection following influenza in a mouse model (Majumder et al., 2024). The licensed OMV-containing vaccine Bexsero provides partial protection against *Neisseria meningitidis* meningitis, though coverage remains limited (Holst et al., 2013). Shehata et al. designed a bivalent OMV-based vaccine against influenza A virus and Middle East Respiratory Syndrome Coronavirus (MERS-CoV), with immunized mice generating high neutralizing antibody titers (Shehata et al., 2019). Martins et al. fused meningococcal OMVs with Zika virus (ZIKV) envelope proteins to form nanoparticle vaccines. These vaccines significantly reduced viral load in infected cells. In the field of respiratory viral vaccines, the application of OMV platforms for RSV vaccine development has also gained exploratory progress. A recent study constructed a synthetic bacterial outer membrane vesicle (SyBV) vaccine displaying the RSV prefusion F protein, which was confirmed to induce high titers of pre-F-specific neutralizing antibodies and confer robust protective immunity against RSV challenge in mouse models (Meng et al., 2025). This work provides direct proof-of-concept that OMV-based delivery platforms are capable of effectively presenting RSV antigens and eliciting protective immune responses, validating the technical feasibility of the OMV-RSV vaccine strategy. Nevertheless, most existing OMV-based RSV vaccine candidates are still derived from pathogenic bacterial strains, which retain potential pro-inflammatory risks and safety concerns in clinical translation, further highlighting the unique value of developing commensal bacteria-derived Bt OMVs as a safer RSV vaccine platform. Additionally, OMVs show promise in cancer vaccines. They can target and accumulate in specific tumor tissues, inducing the production of CXCL10 and interferon-gamma (IFN-γ), thereby inhibiting tumor growth (Kim O. Y. et al., 2017). As drug delivery vehicles, attenuated *Klebsiella pneumoniae* OMVs loaded with doxorubicin inhibited non-small cell lung cancer growth without significant *in vivo* toxicity (Kuerban et al., 2020). Surface expression of targeting ligands on OMVs enables the specific delivery of siRNA to tumor cells (Gujrati et al., 2014). Engineered OMV-PD1 binds to PD-L1 on tumor cells, inducing its endocytosis and degradation, thereby disrupting the PD-1/PD-L1 pathway and significantly inhibiting tumor growth (Li et al., 2020).

Collectively, these studies demonstrate the feasibility of engineering OMVs to induce protective immune responses, laying a foundation for exploring OMVs derived from the human commensal Bt for RSV vaccines. However, the clinical translation of OMV-based vaccines derived from conventional pathogenic bacteria for RSV infection needs to address several challenges: OMVs derived from non-commensal pathogens may elicit unintended toxicities (Velimirov and Velimirov, 2024); the lack of standardized manufacturing protocols leads to significant batch-to-batch variability in OMV composition and size, impacting vaccine quality control (Hong et al., 2019); and larger-scale clinical trials are essential to more accurately assess the safety and efficacy of OMV-based vaccines, particularly studies focusing on their applicability in diverse populations.

## 4 Development potential of Bt OMVs for RSV vaccines

Bt is a highly abundant Gram-negative commensal bacterium in the mammalian gut microbiota (Wong et al., 2024). During its growth cycle, Bt sheds OMVs from its outer membrane. Bt utilizes these OMVs to communicate with the host, fulfilling key functions: reinforcing the intestinal mucosal barrier, modulating the stability of the gut immune system, and facilitating the digestion of host glycans and dietary fiber (Wang X. et al., 2023; Sartorio et al., 2023). Compared to OMVs derived from classical pathogens, Bt OMVs, originating from a commensal bacterium, generally exhibit lower toxicity. This characteristic provides a natural advantage for addressing safety concerns in the application of OMVs, making them particularly suitable as carriers for RSV vaccine development. Bt OMVs not only exhibit a non-pathogenic mutualistic relationship with the host organism but also enhance responses of Treg and Th1 cells while reducing the activation of Th2 and Th17 cells (Gul et al., 2022). The emergence of single-cell sequencing and multi-omics integration technologies enables high-resolution profiling of T cell subset differentiation trajectories and molecular regulatory networks at the single-cell level, providing powerful methodological tools for in-depth dissection of how Bt OMVs shape the T cell immune landscape (Song et al., 2025). This immunomodulatory property is crucial for their utility as vaccine carriers, particularly for avoiding Th2/Th17-dominant inflammatory responses that contribute to vaccine-enhanced disease (VED). Research has shown that Bt alone can induce a humoral response characterized by IgA and IgG production in germ-free mice (Peterson et al., 2015). This suggests that Bt OMVs, as vaccine delivery vehicles, may possess adjuvant-like activity capable of eliciting robust protective immune responses. Carvalho et al. utilized triparental mating to express Salmonella antigens on Bt OMVs. Following intranasal administration, CD103^−^CD11b^+^ dendritic cells in both the upper and lower respiratory mucosa internalized the Bt OMVs within 24 h, thereby initiating T cell responses and generating antigen-specific IgA and IgG antibodies (Carvalho et al., 2019a). Using the same methodology, they constructed an OMV-based vaccine containing the *Yersinia pestis* V antigen, which similarly induced IgA production at mucosal sites and IgG in the serum. Although oral delivery must overcome barriers like the GI tract environment, pH, enzymes, and microbiota, antigen-specific antibody responses were still detectable (Carvalho et al., 2019b). Proteomic profiling of the gastrointestinal tract has revealed that mucosal immune homeostasis is tightly linked to systemic immune function, which provides a physiological basis for gut commensal bacteria-derived OMVs to exert systemic immunomodulatory effects and induce cross-protective immune responses at distal respiratory mucosa via the gut-lung axis (Koriem, 2026). These findings demonstrate the feasibility of Bt OMVs as antigen delivery vehicles and highlight their high immunogenicity and critical role in inducing mucosal immune responses.

Despite the proven feasibility of Bt OMVs as antigen delivery platforms, the loading strategy for the conformation-sensitive RSV pre-F protein remains a core technical challenge that directly determines vaccine immunogenicity. Two dominant antigen-loading approaches for OMV-based vaccines have been developed, each with distinct trade-offs between conformational integrity and immune accessibility.

The first strategy is surface display, typically achieved by fusing the target antigen to outer membrane anchoring proteins such as ClyA or OmpA. This approach enables direct exposure of antigens on the OMV surface, allowing immediate recognition by B cell receptors (BCRs) and thus facilitating robust induction of neutralizing antibodies. This methodology is also compatible with the triparental mating technique widely used for Bt OMV antigen engineering. However, the transmembrane anchoring structure and fusion tag may introduce spatial steric hindrance and mechanical tension, which can disrupt the native trimeric conformation of pre-F protein and trigger its irreversible transition to the post-fusion state. Such conformational loss would eliminate key neutralizing epitopes and severely compromise the protective efficacy of the vaccine.

The second strategy is lumen encapsulation, in which antigens are expressed in the bacterial periplasm and naturally encapsulated during OMV biogenesis, or loaded into the vesicle lumen via ex vitro methods such as electroporation. The lipid bilayer of OMVs provides a physical protective barrier for the cargo antigen, shielding it from enzymatic degradation and environmental interference. This feature is particularly advantageous for preserving the fragile pre-fusion conformation of RSV F protein. Nevertheless, lumen-encapsulated antigens cannot directly engage BCRs and must be internalized, processed, and presented by antigen-presenting cells (e.g., dendritic cells), which may reduce the magnitude and rapidity of the humoral immune response. Additionally, this strategy generally faces limitations such as low loading efficiency and uncontrollable antigen release kinetics. The core characteristics, application advantages, and key technical bottlenecks of the two loading strategies for RSV pre-F protein delivery are systematically compared in Table 2.

**Table 2:** Comparison of two antigen-loading strategies for Bt OMV-based RSV vaccines.

Loading strategy	Technical approach	Advantage for RSV vaccine	Core challenge for pre-F protein
Surface display	Fusion with outer membrane proteins (ClyA, OmpA)	Direct BCR recognition; robust neutralizing antibody induction	Risk of pre-F conformational disruption by anchoring structure
Lumen encapsulation	Periplasmic expression / *in vitro* loading	Superior protection of pre-F native conformation	Reduced BCR accessibility; low loading efficiency

Collectively, the two strategies present an inherent trade-off between antigen conformational stability and immune recognition efficiency, and the customized optimization of loading modalities for RSV F protein represents a critical technical direction for the development of Bt OMV-based RSV vaccines.

**Figure 1:**
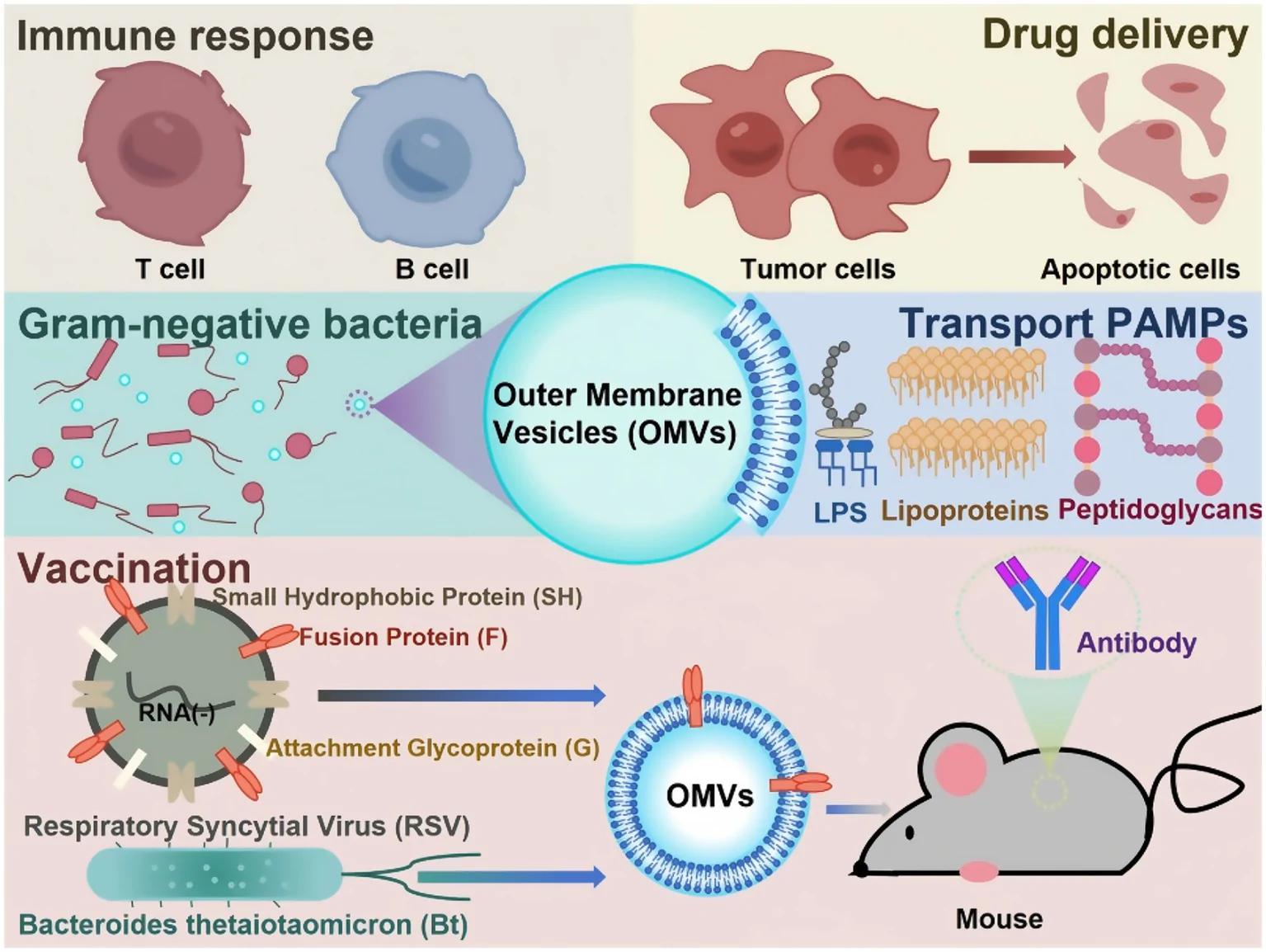
Multifunctional applications of OMVs. (1) OMVs can deliver PAMPs to elicit immune responses; (2) OMVs serve as drug delivery carriers for therapeutic agents in cancer treatment; and (3) OMVs function as vaccine platforms, with the targeted delivery of the RSV F protein via Bt OMVs representing a promising strategy for establishing a novel RSV vaccine.

Furthermore, studies indicate that in inflammatory microenvironments, Bt primarily influences a distinct Treg subtype, RORγt^+^FoxP3^+^ Treg cells, rather than conventional CD4^+^CD25^+^FoxP3^+^ Treg cells. This RORγt^+^ subtype demonstrates enhanced stability and greater anti-inflammatory activity, effectively suppressing excessive Th17 responses (Cantorna et al., 2019; Furuyama et al., 2022; Kim B. S. et al., 2017). The immunomodulatory effects of Bt on T cells are likely mediated by its outer membrane antigens. This is supported by studies using *Bacteroides thetaiotaomicron*-specific CD4^+^T cell hybridomas (BθOM). The transfer of transgenic T cells expressing the BθOM TCR into healthy mice resulted in their proliferation and differentiation into either Tregs or effector T cells within the colon, colonic draining lymph nodes, and spleen. Critically, depletion of Bt-specific Tregs triggered inflammatory responses (Wegorzewska et al., 2019). Given that OMVs are derived from and retain the composition of the outer membrane, constructing an RSV vaccine using Bt OMVs as a carrier platform offers the potential to modulate the balance between T cell subsets and suppress excessive inflammation. This strategy may circumvent the risk of VED (Figure 1).

## 5 Outlook

The high prevalence of RSV and its associated disease burden underscore the urgent need to develop safe and highly efficacious RSV vaccines. Although significant progress has been made in RSV vaccine development in recent years, exemplified by the approval of pre-F protein-targeted vaccines, challenges persist, including suboptimal immunogenicity and safety concerns. OMVs, leveraging their inherent adjuvant properties, represent an emerging direction in vaccine development. However, OMVs derived from classical pathogens face limitations due to inherent pro-inflammatory risks and challenges in production standardization. In contrast, Bt OMVs provide a novel and promising solution to overcome these limitations, demonstrating unique value in reducing pro-inflammatory risks while maintaining robust immune activation. The low inflammatory potential of Bt OMVs and their ability to modulate T cell subsets may circumvent the risk of VED associated with traditional vaccines. Furthermore, Bt OMVs can deliver antigens via mucosal routes (e.g., intranasal or oral administration), effectively stimulating local mucosal immune responses. This capability holds promise for addressing the insufficient mucosal immunity elicited by current injectable vaccines.

Therefore, the innovative strategy of utilizing Bt OMVs as carriers for the targeted delivery of the RSV F antigen aims to establish a novel RSV vaccine platform offering enhanced safety and efficacy. This approach heralds the potential for the development of safer, more effective, and more accessible RSV prophylactics, contributing significantly to global public health. Future research should focus on: (1) Optimizing the expression and delivery strategies of the RSV F antigen within Bt OMVs to ensure antigen stability and potent immunogenicity. For surface display strategies, priority should be given to screening suitable outer membrane anchoring scaffolds and designing flexible polypeptide linkers to minimize conformational disruption of the pre-F protein while maximizing antigen surface exposure. For lumen encapsulation strategies, research should focus on improving antigen loading efficiency and establishing controllable endosomal release mechanisms to balance conformational protection and immune presentation efficiency. High-throughput quantitative proteomics can be applied to dynamically monitor antigen loading efficiency and OMV cargo composition, providing data support for process optimization; (2) Conducting an in-depth investigation into the biological properties and immunomodulatory mechanisms of Bt OMVs to ensure vaccine safety through precise compositional control. An integrated proteomics and metabolomics approach can be adopted to screen differentially expressed proteins and metabolites after Bt OMV intervention, map the key signaling pathways of immunomodulation, and systematically elucidate the molecular mechanism by which Bt OMVs regulate T cell subset balance (Li et al., 2025); (3) Evaluating the safety, immunogenicity, and efficacy of the Bt OMV-based RSV vaccine in animal models. Differential proteomics can be utilized to identify key host proteins and pathways modulated by vaccination, screen potential immune biomarkers associated with protective efficacy, and validate the capacity of the vaccine to induce durable immunological memory to provide robust, long-term protection against RSV infection (Lan et al., 2025); and (4) Exploring combinatorial formulation strategies to further augment vaccine immunogenicity. For intranasal or oral mucosal administration, Bt OMVs can be formulated with mucoadhesive polymers (e.g., chitosan, hyaluronic acid) to prolong retention on the respiratory or gastrointestinal mucosal surface, enhance antigen uptake by local dendritic cells, and amplify mucosal IgA responses. In addition, Bt OMVs can be co-delivered with well-characterized immunostimulatory molecules such as selective TLR agonists or cytokine adjuvants in a dose-controlled manner. This combinatorial design can further strengthen innate immune priming and promote the differentiation of effector T cells and long-lived memory B cells, while preserving the inherent low-inflammatory property and Treg-modulating activity of Bt OMVs to maintain a favorable balance between protective efficacy and safety.
